# Simple, high-throughput measurement of gut-derived short-chain fatty acids in clinically relevant biofluids using gas chromatography-mass spectrometry

**DOI:** 10.1016/j.jmsacl.2022.07.002

**Published:** 2022-07-16

**Authors:** Joshua T Bain, Maarten W Taal, Nicholas M Selby, James C Reynolds, Liam M Heaney

**Affiliations:** a School of Sport, Exercise & Health Sciences, Loughborough University, Loughborough, UK; b Centre for Kidney Research and Innovation, Academic Unit for Translational Medical Sciences, School of Medicine, University of Nottingham, Nottingham, UK; c Renal Unit, University Hospitals of Derby and Burton NHS Foundation Trust, Derby, UK; d Centre for Analytical Science, Department of Chemistry, Loughborough University, Loughborough, UK

**Keywords:** Biomarker, Gas chromatography-mass spectrometry, Kidney disease, Short-chain fatty acids, Validation

## Abstract

**Introduction:**

The quantitative measurement of circulating gut bacteria-derived metabolites has increased in recent years due to their associations with health and disease. While much of the previous attention has been placed on metabolites considered as deleterious to health, a shift to the investigation of short-chain fatty acids (SCFAs) as potential health promotors has been observed.

**Objectives:**

To develop a simple, high-throughput and quantitative assay to measure gut-derived SCFAs in clinically relevant biofluids using gas chromatography-mass spectrometry (GC–MS).

**Methods:**

A short (7.5 min) GC–MS assay was optimized for measurement of seven straight- and branched-chain SCFAs and their deuterated isotopes using a wax-based column for analysis without prior derivatization. The assay was validated using routine criteria to assess precision, accuracy, matrix effects, recovery, and extraction reproducibility. Assay applicability was tested in cohorts of healthy individuals and kidney disease patients.

**Results:**

The assay was demonstrated to be precise, accurate and reproducible with acceptable levels of matrix effect and analyte recovery. Lower limits of detection and quantitation were in the low ng/mL range. An investigation into different blood collection tube chemistries demonstrated that lithium heparin plasma and serum clotting activator tubes are recommended for use in future cross-study comparisons. Kidney disease patient analyses demonstrated variable differences across SCFAs when comparing hemodialysis to earlier stages of chronic kidney disease, demonstrating the suitability of the assay for translation to clinical analyses.

**Conclusion:**

The assay has been validated and identified as reliable for use in larger-scale studies for the analysis of SCFAs in human plasma and serum.

## Highlights

• A high-throughput assay for measurement of short-chain fatty acids in biofluids.
• A simple, time efficient liquid–liquid extraction protocol with no derivatization.
• Recommend use of lithium heparin plasma or clotting activator serum collection.
• Clinical applicability demonstrated by measurement of kidney disease patient samples.

## Nomenclature

CKD: chronic kidney disease
DBS: dried blood spot
EDTA: K_3_ ethylenediaminetetraacetic acid
ESKD: end-stage kidney disease
GC–MS: gas chromatography-mass spectrometry
eGFR: estimated glomerular filtration rate
IS: internal standard
iTrend: Intelligent Technologies for Renal Dialysis
LiHep: lithium heparin
LLOD: lower limit of detection
LLOQ: lower limit of quantitation
ME: matrix effect
MTBE: methyl tertbutyl ether
QC: quality control
RRID: Renal Risk in Derby
RSD: relative standard deviation
SCFAs: short-chain fatty acids
Serum: serum clotting activator
S-Gel: serum gel separator
SIM: selected ion monitoring
VAMS: volumetric absorptive microsampling

## 1 Introduction

In recent years, the quantitative measurement of circulating gut-derived metabolites has become an area of increasing interest as studies have identified associations between the concentrations of bacterial metabolites and the progression of chronic conditions [1]. These metabolites originate from multiple bacterial metabolic pathways, with the metabolism of dietary products, such as quaternary amine compounds (e.g. choline and l-carnitine) and amino acids (e.g. tyrosine and l-tryptophan), in the gastrointestinal tract. These processes produce downstream metabolites such as trimethylamine *N*-oxide, p-cresol, and indoxyl sulfate, all of which have been shown to associate with the progression of chronic disease [1].

In contrast, beneficial effects have been associated with other gut bacteria-derived metabolites, particularly short-chain fatty acids (SCFAs). SCFAs exist as straight or branched-chain molecules, containing less than six carbon atoms and a single carboxyl group and are produced predominantly by the bacterial fermentation of non-digestible dietary fibre [1]. They have been observed to provide a protective/therapeutic effect for gastrointestinal conditions [2], [3]. While the beneficial effects of SCFAs have been well documented for gut-related disorders [4], emerging evidence shows the presence/manipulation of SCFAs demonstrates positive effects on chronic and acute conditions not related to the gastrointestinal system [5], [6]. These effects may be mediated via anti-inflammatory response induced by SCFAs, which has been shown to blunt fibrotic, inflammatory and oxidative stress mechanisms [7], and improve clinical outcomes in animal models of disease [8].

These positive findings offer the potential of circulating SCFAs to be measured as clinical biomarkers. However, in order to apply these assessments to clinical situations a precise, cost-effective, and easy-to-translate assay is necessary. Modern analytical techniques for the analysis of SCFAs rely on the use of chromatographic separation techniques coupled with mass spectrometry [1]. However, many of the current methods available require a derivatization step and/or lack the necessary throughput required for clinical translation [9], [10], [11]. These assay characteristics lead to increased sample preparation time and cost, as well as often requiring the use of hazardous chemicals to achieve optimal derivatization. The current experiment sought to develop a reproducible, high-throughput, sensitive, and comparably cost-effective SCFA assay for use with clinically relevant biofluids, and validate it using routine bioanalytical validation criteria [12], [13].

## 2 Materials and methods

### 2.1 Materials

**Fig. 1:**
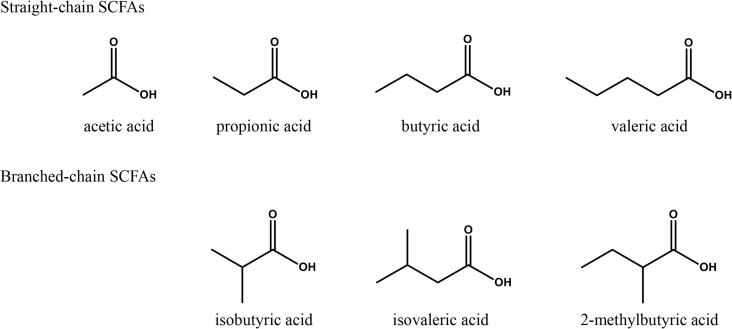
Chemical structure and terminology for the seven straight- and branched-chain short-chain fatty acids (SCFAs) measured within the assay.

Acetic acid (≥99.99 % purity), propionic acid (≥99.5 % purity), isobutyric acid (≥99.5 % purity), butyric acid (>99 % purity), isovaleric acid (>99 % purity), 2-methylbutyric acid (>99 % purity), and valeric acid (≥99.8 % purity) were purchased from Merck (Gillingham, UK). Acetic acid‑d_4_ (≥99.5 % purity, >95 % isotopic enrichment) was purchased from Alfa Aesar (Heysham, UK), isobutyric acid-d3 (98.7 % purity, >95 % isotopic enrichment) was purchased from QMX (Thaxted, UK), propionic acid-d3 (98 % purity, 99.6 % isotopic enrichment), butyric acid-d7 (98 % purity, 99.3 % isotopic enrichment), isovaleric acid-d9 (98 % purity, 97.9 % isotopic enrichment), 2-methylbutyric acid-d3 (99 % purity, 99.3 % isotopic enrichment) and valeric acid-d9 (98 % purity, 98.6 % isotopic enrichment) were purchased from Toronto Research Chemicals (Toronto, Canada). Methyl tertbutyl ether (MTBE) (99.9 % purity) was purchased from Acros Organics (Loughborough, UK). LC-MS grade water and hydrochloric acid (1 M) were purchased from VWR Chemicals (Lutterworth, UK). All SCFAs included within the assay are detailed in Fig. 1.

### 2.2 Human participants

In order to compare the use of different blood collection methods, blood samples were collected from an antecubital vein into blood collection tubes from 51 healthy participants (median (range); 26 (19–55) yrs; 69 % male). Urine was also collected within 15 min prior to venous draw. Following collection, urine and plasma samples were immediately separated by centrifugation at 2500×*g*, 4 °C for 20 min and transferred to aliquots for storage at −80 °C. Serum samples were allowed to clot while placed on ice for 30 min prior to centrifugation under the same conditions. All human participants were provided with details of the research study in plain English and provided written informed consent. Participants were able to withdraw their consent at any point without reason and all participant data were anonymized at the earliest opportunity. All relevant study protocols complied with the Declaration of Helsinki and were approved by the local ethics committee.

Clinical patient samples were collected as part of the Royal Derby Hospital Renal Unit Intelligent Technologies for Renal Dialysis (iTrend) program and Renal Risk in Derby (RRID) study [14], [15]. Thirty-five end-stage kidney disease (ESKD) patients (69 (42–90) yrs; 54 % male) from the iTrend study with serum samples collected shortly before a hemodialysis session were included alongside two age- and sex-matched chronic kidney disease (CKD) patient groups from the RRID study. The CKD patients were split by an estimated glomerular filtration rate (eGFR) of < 60 (34.1 (23.4–54.1) mL/min/1.73 m^2^) or ≥ 60 (68.5 (60.3–84.3) mL/min/1.73 m^2^). Each study was individually approved by the Health Research Authority Research Ethics Committee and abided by the principles of the Declaration of Helsinki. All participants provided written informed consent.

### 2.3 Sample preparation

All samples were prepared for analysis via liquid–liquid extraction by mixing 100 µL of an internal standard (IS) mixture containing 6 µg/mL of each deuterium-labelled SCFA in MTBE with 100 µL of the biofluid sample and 100 µL of 1 M hydrochloric acid. The mixture was vortexed for 30 s to ensure complete mixing. The resultant mixture was centrifuged at 2500×*g*, 4 °C for 15 min to separate the organic (MTBE) and aqueous layers. The organic layer (∼100 µL) was then transferred to a low volume crimp top autosampler vial for analysis. Test samples used for recovery, matrix interference and reproducibility experiments were produced by mixing eight randomly chosen samples of each sample matrix from different healthy individual participants.

### 2.4 Sample analysis

**Fig. 2:**
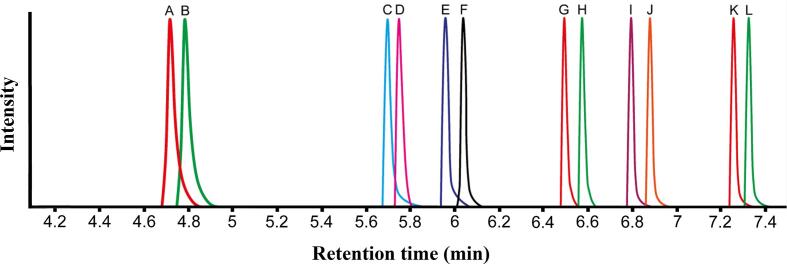
Base peak intensity chromatogram of seven straight- and branched-chain short-chain fatty acids and their labelled isotopes by gas chromatography-single quadrupole mass spectrometry. (A) acetic acid‑d_4_, *m*/*z* 63 (B) acetic acid, *m*/*z* 60 (C) propionic acid-d3, *m*/*z* 77 (D) propionic acid, *m*/*z* 74 (E) isobutyric acid-d3, *m*/*z* 76 (F) isobutyric acid, *m*/*z* 73 (G) butyric acid-d7, *m*/*z* 63 (H) butyric acid, *m*/*z* 60 (I) isovaleric acid-d9, *m*/*z* 63, 2-methylbutyric acid-d3, 79 *m*/*z* (J) isovaleric acid, *m*/*z* 60, 2-methylbutyric acid, *m*/*z* 74 (K) valeric acid-d9, *m*/*z* 63 (L) valeric acid, *m*/*z* 60.

Samples were analyzed in duplicate by gas chromatography-mass spectroscopy (GC–MS) using a scheduled selected ion monitoring (SIM) protocol on a single quadruple mass analyzer. A 7820A GC system coupled with a 5977B MSD (both Agilent Technologies, Stockport, UK) was used to perform all analyses. The GC system was fitted with a Stabilwax-DA Crossbond Carbowax PEG column (30 m × 0.5 mm × 0.25 µm; Thames Restek, High Wycombe, UK). Electron ionization was applied with a fixed ionization energy of 70 eV, and the source temperature was set to 230 °C. The quadrupole mass analyzer temperature was set to 150 °C. The injector inlet and transfer line temperature were set to 250 °C, with injection volume set to 3 µL and the split ratio at 5:1 with a flow of 10 mL/min. Purified helium was used as a carrier gas and had a constant flow rate of 2 mL/min. The GC oven temperature cycle was programmed as follows: initial temperature 80 °C and held for 1 min, increased linearly to 127 °C at 10 °C/min, and then increased linearly to 181 °C at 30 °C/min. The total run time was 7.5 min followed by a post-run temperature hold of 230 °C for 2 min. The approximate injection-to-injection time period was 12.5 min. The MS detector was set to scheduled SIM mode as detailed in Table 1. An example base peak intensity chromatogram of all SCFAs and their corresponding IS is shown in Fig. 2. Mass Hunter software (Version B.07.00; Agilent Technologies, Stockport, UK) was used for GC–MS data acquisition and processing.

**Table 1:** Scheduled selected ion monitoring (SIM) parameters for measurement of seven straight- and branched-chain short-chain fatty acids and their labelled isotopes by gas chromatography-single quadrupole mass spectrometry.

SIM group	SIM window (min)	Analyte	Ion (*m*/*z*)	Dwell time (ms)
1	4.00–5.50	acetic acid	60	156
		acetic acid-d_4_	63	16
2	5.50–5.85	propionic acid	74	162
		propionic acid-d3	77	16
3	5.85–6.20	isobutyric acid	73	178
		isobutyric acid-d3	76	18
4	6.20–6.65	butyric acid	60	89
		butyric acid-d7	63	9
5	6.65–7.00	isovaleric acid	60	78
		isovaleric acid-d9	63	8
		2-methylbutyric acid	74	78
		2-methylbutyric acid-d3	79	8
6	7.00–7.50	valeric acid	60	72
		valeric acid-d9	63	7

### 2.5 Assay calibration, precision, and accuracy

Calibration standards were prepared via serial dilution of SCFAs in water to concentrations of 40, 60, 100, 200, 300, 400 and 500 ng/mL. All analytes were calibrated on this scale, except acetic acid, which was calibrated at 10-fold increased levels due to its increased levels in biosamples (400, 600, 1000, 2000, 3000, 4000 and 5000 ng/mL). Analytical preparation of calibration standards was performed using an identical approach to sample preparation and analyses were performed in triplicate. Calibration equations were produced by comparing the known concentrations against the response ratio of each IS and its corresponding analyte. The linearity of each calibration experiment was calculated using least squares regression. All injections were preceded by a blank injection (100 % MTBE) to ensure no carryover occurred between standards/samples. An additional calibration to understand the upper constraints of the assay was performed to test linearity up to 16x higher (i.e. 8000/80000 ng/mL) than the standard calibration range.

The lower limit of quantitation (LLOQ) was determined by identifying the lowest concentration that provided a response of at least five times that observed in a blank measurement and had a reproducibility (relative standard deviation [RSD] of repeated injections) of ≤ 20 %. This was identified as 40 ng/mL for each analyte, except acetic acid, which was not tested for reproducibility below 400 ng/mL due to it being known to be present at high levels in biofluid samples. The theoretical lower limit of detection (LLOD) was mathematically calculated as the response corresponding to at least three times that seen from blank injections.

In order to assess inter- and intra-day precision, quality control (QC) standards were produced at levels corresponding to the LLOQ (40/400 ng/mL for SCFAs and acetic acid, respectively), as well as a low (75/750 ng/mL), medium (200/2000 ng/mL) and high (375/3750 ng/mL) biofluid sample. QC standards were prepared in a similar fashion to calibration standards via serial dilution of SCFAs in water and subjected to the described sample extraction protocol. Inter-day precision was assessed by performing duplicate injections of each QC a total of three times per day across three consecutive days. In addition, intra-day precision was assessed by duplicate analysis of each QC standard repeated six times within a 24-hr period. Analytical precision was determined by evaluating the RSD of response ratios for each SCFA at each QC level.

Accuracy of the assay was assessed by comparing the calculated concentrations of the low, medium, and high QC standards and comparing these to the expected concentrations.

### 2.6 Matrix effect and recovery

Matrix effect (ME) and assay recovery were assessed for each matrix of interest. Different matrices of interest included plasma collected via blood tubes containing K_3_ ethylenediaminetetraacetic acid (EDTA) and lithium heparin (LiHep), serum samples collected via blood tubes containing a clot activator (Serum) and a serum gel separator (S-Gel) (all S-monovette; Startstedt, Leicester UK), and neat urine. ME was determined by calculating the differences between the computed calibration gradient of the neat SCFAs in water against concentration-matched spikes of SCFAs in the mixed test sample for each matrix [16].

Analyte recovery was assessed by calculating the difference in concentration between a pre- and post-extraction SCFA spiked test sample at levels corresponding to the low, medium, and high QC standards.

### 2.7 Reproducibility of sample extraction

Reproducibility of sample extraction was assessed across all matrices by extracting SCFAs from the test samples a total of five separate times. All extractions were run in duplicate, and RSDs were determined for the calculated concentration across all injections for each matrix.

### 2.8 Comparison of biofluid collection methods

Blood samples were collected in S-monovette tubes containing EDTA, LiHep, Serum or S-Gel modifiers. All blood samples tubes were drawn in the order according to manufacturer recommendations. To assess measured SCFA concentrations across collection methods, samples collected from all 51 healthy individuals were analyzed and compared. Variation in concentrations measured for each SCFA were analyzed using a Related-Samples Friedman’s Two-Way Analysis of Variance by Ranks with a Wilcoxon post-hoc test for multiple comparisons. The potential for urine samples to be employed as a non-invasive collection technique was investigated by assessing Spearman’s rho (r) correlations of urine measurements to each blood measurement. An α (p) value of < 0.05 was employed and statistical analyses were performed using RStudio (v.1.2.5042). All samples where a peak was detected, but fell below the LLOQ, were recorded at the LLOD value.

### 2.9 Clinical application

To assess clinical applicability, serum samples from the iTrend (ESKD) and RRID (CKD) studies were analyzed for SCFA levels using the described method. Distribution of SCFAs across the three age- and sex-matched groups (CKD eGFR ≥ 60 vs CKD eGFR < 60 vs ESKD prior to hemodialysis) were compared using a two-way Mann-Whitney *U* test. All samples where a peak was detected, but fell below the LLOQ, were recorded at the LLOD value.

## 3 Results

### 3.1 Assay calibration, precision, and accuracy

Carryover was confirmed not to be present, as not one blank injection contained any SCFA peaks above 20 % of the LLOQ or IS peaks above 5 % of the expected level. All calibration experiments produced a correlation coefficient (r^2^) of ≥ 0.993. LLODs were calculated at 28.6 ng/mL (acetic acid), 20.5 ng/mL (propionic acid and valeric acid), 17.3 ng/mL (isovaleric acid), 14.9 ng/mL (isobutyric acid), 12.3 ng/mL (butyric acid), and 9.6 ng/mL (2-methylbutyric acid). The assay remained linear for all SCFAs at the extended calibration range (40–8000/400–80000 ng/mL) with r^2^ values reported as ≥ 0.999.

Inter-day precision of QC analyses across all SCFAs was ≤ 9.2 % with an intra-day precision of ≤ 5.0 %. The assay was confirmed to be sufficiently accurate, as no calculated QC concentration deviated by > 20 % of the expected value. **Table S1** of the supplementary information provides the detailed results for each individual SCFA. **Figure S1** shows the raw SIM chromatograms of each SCFA for a representative medium QC sample.

### 3.2 Matrix interference and recovery

The observed ME ranged from 71 to 137 %. Specifically, EDTA, LiHep and Serum matrices displayed the least interference with ME values from 82 to 103 %. Mean recovery across all matrices and SCFAs was 90 %. Recovery levels were observed to decrease with decreasing acid chain length. This is due to an increasing molecule polarity as alkyl chains shorten, which reduces the partition into the MTBE (organic) layer. Table 2 displays a summary of ME and recovery data across the three major SCFAs of acetic acid, propionic acid, and butyric acid. The remaining values for additional SCFAs are included in **Table S2**.

**Table 2:** Recovery and matrix effect (ME) values for acetic acid, propionic acid and butyric acid analyzed across different matrices in low (L), medium (M) and high (H) quality control (QC) standards.

	acetic acid				propionic acid				butyric acid			
		Recovery (%)				Recovery (%)				Recovery (%)		
	ME	L QC	M QC	H QC	ME	L QC	M QC	H QC	ME	L QC	M QC	H QC
**EDTA**	93	46	29	18	99	95	87	62	95	114	113	83
**LiHep**	88	82	54	36	103	91	71	58	94	111	92	80
**Serum**	90	66	60	40	99	74	82	64	97	112	108	88
**S-Gel**	72	53	57	40	85	71	72	69	81	87	102	102
**Urine**	120	73	57	35	137	99	86	63	101	117	116	98

EDTA = ethylenediaminetetraacetic acid plasma; LiHep = lithium heparin plasma; Serum = clotting activator serum; S-Gel = serum gel separator.

### 3.3 Reproducibility of sample extraction

SCFAs were detected in all test samples. Valeric acid was detected at a level below the LLOQ and, therefore, the reproducibility of extraction data refers to the raw response ratio values and has not been converted to a quantitative value. The RSDs of multiple extractions ranged from 2 to 14 % demonstrating a reproducible extraction across all SCFAs and matrices. A summary of results is provided in **Table S3.**

### 3.4 Comparison of biofluid collection methods

**Fig. 3:**
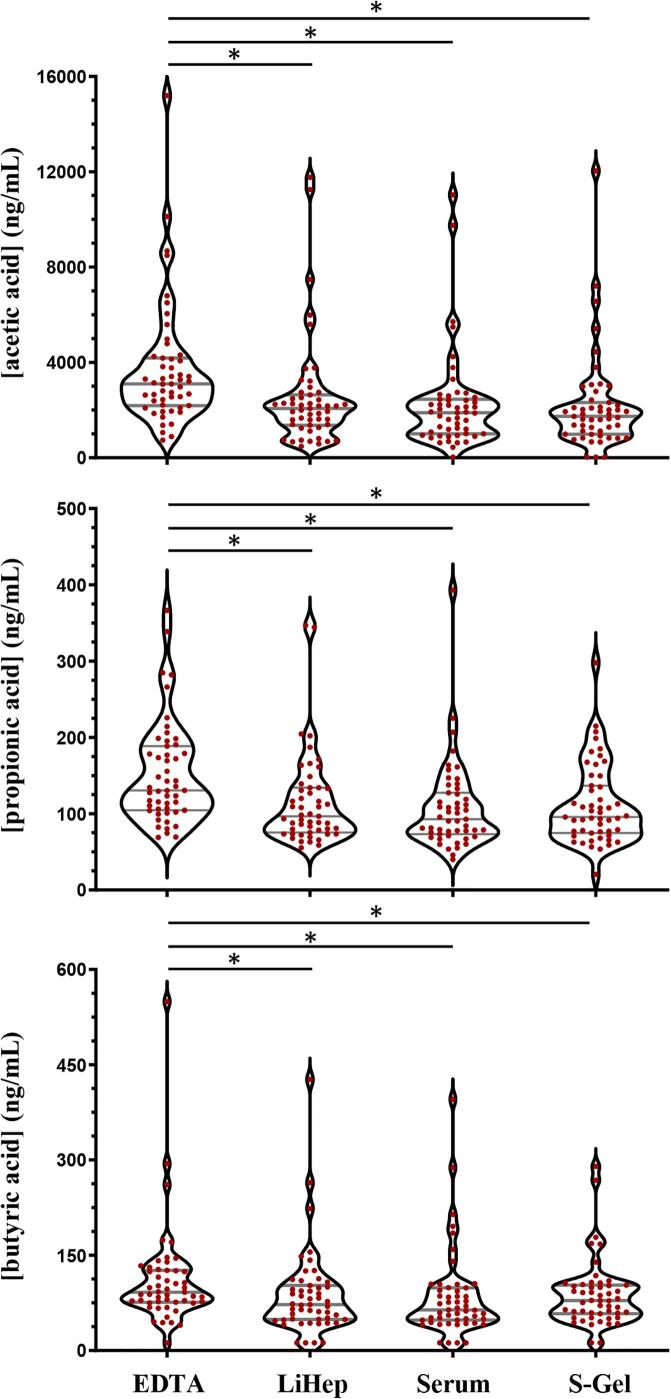
Violin plots to compare acetic acid (top), propionic acid (middle) and butyric acid (bottom) measurements from 51 healthy participants across different blood collection methods. EDTA = ethylenediaminetetraacetic acid plasma; LiHep = lithium heparin plasma; Serum = clotting activator serum; S-Gel = serum gel separator. * Denotes p < 0.05.

Friedman’s two-way analysis of variance by ranks with a Wilcoxon post-hoc test reported that LiHep, Serum, and S-Gel did not differ from each other across all SCFAs, demonstrating these collection modalities as most efficient for comparison across experiments (see Fig. 3 **and Figure S2**). Valeric acid was detected in all samples, but no individual sample demonstrated a level greater than or equal to the LLOQ.

Correlation experiments to compare urine to other collections methods demonstrated a lack of validity for urine as an alternative non-invasive collection method. Spearman’s rho correlation coefficients (r) for isobutyric acid (0.39–0.63) showed reasonable correlations, with all other SCFAs showing either no comparative characteristics or levels in urine below the LLOQ. Despite a reasonable correlation for urine measurements for isobutyric acid, only 63 % of urine samples had values above the LLOQ. Calculated r values comparing urine to all other collection methods are shown in **Table S4**.

### 3.5 Clinical application

**Fig. 4:**
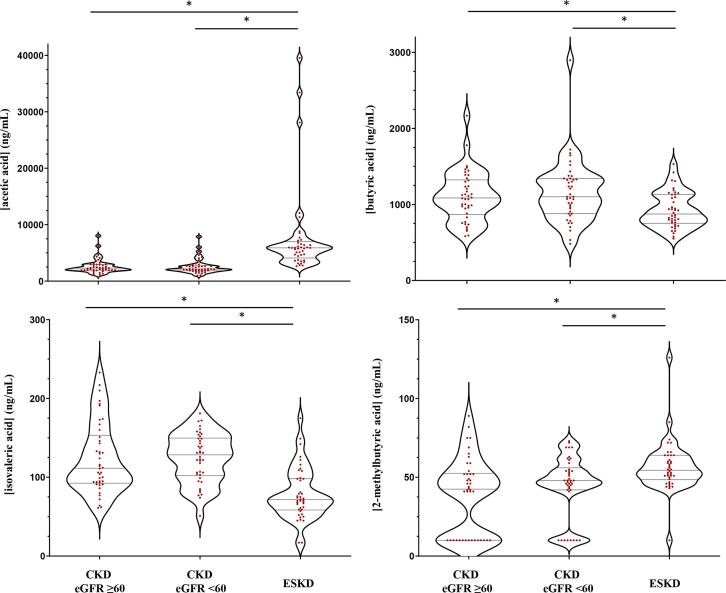
Violin plots to show the distribution of acetic acid (top left), butyric acid (top right), isovaleric acid (bottom left) and 2-methylbutyric acid (bottom right) concentrations in chronic kidney disease (CKD) and end-stage kidney disease (ESKD) patient serum. * Denotes p < 0.05.

In order to assess the clinical application of the validated assay, a series of CKD and ESKD patient samples were analyzed for the seven SCFAs. In general, SCFA concentrations in kidney disease patients were increased when compared to healthy participant measurements, albeit the samples were not age- and sex-matched. Two SCFAs, isovaleric acid and 2-methylbutyric acid, demonstrated a general decrease in concentrations in the kidney patient cohorts when compared to healthy comparators. When assessing differences between kidney disease patient groups, ESKD patients showed increased levels of acetic acid and 2-methlybutyric acid, but lower levels of butyric acid and isovaleric acid (Fig. 4). No differences were seen between patient cohorts for propionic acid and isobutyric acid, and although valeric acid was increased in ESKD patients, only approximately 30 % of analyzed samples had levels above the LLOQ (**Figure S3**). **Figure S4** shows the raw SIM chromatograms of each SCFA for a representative ESKD patient.

## 4 Conclusions

The present study describes a simple, high-throughput protocol to quantitatively analyze seven straight- and branched-chain SCFAs by GC–MS. The assay is precise, accurate, and offers a wide dynamic range of analysis with linearity confirmed across ng/mL to µg/mL levels. A scheduled SIM protocol is utilized in order to maximize sensitivity, and compound-matched deuterated internal standards are included to ensure the reproducibility required for longer-term large-scale studies. The inclusion of the full range of internal standards also allows for more likely success in translation across locations and equipment. A straight-forward liquid–liquid extraction provides rapid sample preparation with high levels of reproducibility and reduced burden on laboratory personnel. The assay was tested across a range of relevant biofluid matrices, and a small clinical investigation was performed to examine method applicability to clinical research populations.

With the increasing interest in gut-derived metabolites, and notably for the measurement of SCFAs, there has been a need to provide clinically relevant assays to facilitate research into associations with disease [1]. Historically, SCFAs have been measured in fecal content where concentrations are generally high [17], and while more recently there has been increased interest in the measurement of circulating SCFA levels, which poses a greater challenge due to their presence at lower concentrations [9]. The described assay provides sufficient sensitivity to measure SCFAs in the low ng/mL level with only the use of entry-grade single quadrupole GC–MS equipment. This offers a cost-effective option for translation to testing laboratories, negating the requirement for expensive equipment purchases and additional technical staff training. The suitability for use with entry-grade equipment also allows facile translation to GC–MS equipment currently employed across many healthcare systems used in the measurement of inborn errors of metabolism [18]. It is noted that the procurement of the full range of deuterated standards creates an increased cost, however, the careful preparation and ultra-cold storage of these in neat solvents allows for repeated use over long periods. It is also feasible for laboratories to include only the relevant internal standards should the targeting of a reduced range of SCFAs be warranted. The use of a wax-based GC column also allows the measurements to be performed without a derivatization step. This decreases the overall time-burden on laboratory staff and reduces the likelihood of exposure to hazardous chemicals often required for derivatization protocols associated with alternative GC–MS and liquid chromatography-MS methods. In addition, the simple liquid–liquid extraction protocol provides feasibility for automation using robotic handling devices [19]. A recently published method for analysis of SCFAs used a similar non-derivatized analysis approach [20]. The method described within this manuscript provides advantages over the Rohde et al. protocol through the presence of additional SCFAs, a corresponding labelled IS for all analytes present within the analysis, the lack of an evaporation step for sample preparation, a decreased total run time (9.5 min vs ∼ 13 min), and increased LLOQ across all comparable SCFAs.

Previous research has highlighted inconsistencies in the measurement of circulating SCFAs when using different blood collection modalities [21]. This was particularly notable for increases in reported acetic acid levels when EDTA anticoagulation was used, presumably generated from a release of acetate ions during the breakdown of EDTA. In this study, a cohort of young and healthy participants volunteered to provide blood samples across multiple blood collection chemistries, alongside a time-matched urine sample. This was completed in a larger cohort size than done previously and replicated the observation of increased acetic acid in EDTA samples, with an approximate 1.6-fold increase in mean cohort reported values (3693 vs 2281 ng/mL for EDTA vs other modalities combined). Interestingly, the analyses reported that concentration differences were not present for any SCFA when comparing LiHep, Serum and S-Gel tubes, demonstrating a reliable approach for comparing sample concentrations between serum and plasma across multiple collections/studies. Taking into account additional information relating to ME and recovery, it is recommended that lithium heparin plasma or serum clotting activator tubes are considered for the prospective planning of any study wishing to quantitate circulating SCFAs. However, S-Gel tube investigations are suitable where analyses are taking place on retrospectively collected cohorts. The investigation for the use of urine as an alternative, and non-invasive, biospecimen for SCFA measurement showed a lack of comparative values across the majority of SCFAs and is, therefore, not recommended. Future work of interest would include the potential use of volumetric absorptive microsampling (VAMS) and dried blood spots (DBS) for collection outside of the clinical/laboratory space (e.g., for remote and/or repeated sampling) [22]. The current study did not investigate application of the assay on fecal sampling protocols. While fecal sampling has been a mainstay approach to assess microbiome-associated SCFA generation, this study intended to measure circulating levels, as these may demonstrate a more biochemical interaction with physiology related to health and disease [23]. Nonetheless, the inclusion of appropriate fecal SCFA extraction methods into a liquid solvent could then be applied to the described method and provides a natural evolution of assay applicability.

Finally, in order to assess the applicability of the current assay to clinical populations, a clinical investigation into kidney disease patients was performed. Kidney disease has garnered a particular interest surrounding the topic of SCFAs and disease, principally owing to the demonstrated anti-inflammatory activity of SCFAs in experimental conditions. Examples of these include *in vitro* demonstration of blunting of inflammatory process [24], [25], as well as studies performed *in vivo* that observed a reduction in markers of kidney damage following acute kidney injury [6], [26]. The current data demonstrated that increased concentrations of three SCFAs were identified in ESKD patients prior to hemodialysis, with two SCFAs reduced and two without differences. These variances may be due to the differences in kidney function. However, the observation of varying directions in differences of SCFA levels between CKD and ESKD patients suggests that multiple factors are likely to be influencing circulating concentrations. This could include the alteration of diet and/or medication in changing both the availability of precursor molecules (i.e., fibrous material), as well as a shift in microbiome diversity [27]. Furthermore, dialysis patients are known to possess a number of aspects of disordered physiology, such as increased inflammation and fluid overload, as well as being exposed to the hemodynamic effects of dialysis treatment [28], [29], [30]. These processes could lead to gut edema and increases in bowel wall permeability, which may directly affect the translocation of SCFAs from the gut into the circulation. This investigation provided a small-scale, cross-sectional set of data to confirm assay applicability for clinical populations; however, it is necessary that larger, longitudinal investigations are performed to adequately understand the associations of SCFAs with disease severity and/or progression.

In conclusion, we have demonstrated a simple, high-throughput, and validated GC–MS assay suitable for the measurement of SCFAs across multiple clinically relevant biofluid matrices. A recommendation for the use of LiHep or Serum tubes is provided, and the application of serum analyses has been demonstrated in clinically relevant cohorts.

## Institutional review board

Ethics Committee approval was obtained for all studies involving human participants.

## Funding sources

JTB was supported by an Elite Athlete Scholarship for PhD study by the Loughborough University Doctoral College. The funding source had no involvement in this research.

## Declaration of Competing Interest

The authors declare that they have no known competing financial interests or personal relationships that could have appeared to influence the work reported in this paper.
